# Perinatal mental health in Serbia: Awareness and attitudes among healthcare providers

**DOI:** 10.1192/j.eurpsy.2026.10753

**Published:** 2026-07-31

**Authors:** M. Marković, D. Mladenović, J. Stojanov, N. Mićanović, I. Jovanović, S. Kostić, M. Petronijević, S. Vrzić Petronijević

**Affiliations:** 1 Clinic for Psychiatry, University Clinical Centre of Serbia, Belgrade; 2 Special Hospital for Psychiatric Disorders “Kovin”, Kovin; 3 Special Hospital for Psychiatric Disorders “Gornja Toponica”, Niš; 4 Department for Gynecology, Health system “Euromedik”; 5 Clinic for Gynecology and Obstetrics, University Clinical Centre of Serbia; 6 School of Medicine, University of Belgrade, Belgrade, Serbia

## Abstract

**Introduction:**

In Serbia, no nationally representative data exist on perinatal depression prevalence, nor are there screening protocols or comprehensive guidelines for professionals caring for pregnant and postpartum women. Awareness, attitudes, and practices remain insufficiently studied. The aim was to identify educational gaps, systemic barriers, and institutional deficiencies in perinatal mental health.

**Objectives:**

The aim was to identify educational gaps, systemic barriers, and institutional deficiencies in perinatal mental health.

**Methods:**

A cross-sectional survey was conducted among gynecology/obstetrics, psychiatry, pediatrics, and nursing staff from public and private healthcare institutions in Belgrade (centra Serbia) and Niš (Southern Serbia), across primary, secondary, and tertiary levels. An anonymous 23-item questionnaire collected demographics, perceptions of pregnancy and postpartum mental health, attitudes toward screening, familiarity with guidelines, therapeutic confidence, and perceptions of institutional support.

**Results:**

Overall, 221 healthcare professionals participated, with mean age 41.2 years and mean experience 13.6 years, including 48 men and 173 women. Most respondents (85.5%, n=189) considered both pregnancy and postpartum a sensitive period with increased risk of mental disorders, with psychiatrists more likely to emphasize the vulnerability of the postpartum period (78,3%, n=54, p<0.01). 17.4% of psychiatrists (n=12) considered pregnancy a protective factor against the onset of mental disorders, while 20.8% of midwives (n=5) and 33.3% of visiting pediatric nurses (n=7) believed there was no difference in vulnerability between the pre-pregnancy and pregnancy periods. The majority supported routine screening for mood disorders during the peripartum period, regardless of profession (≥85%). Large gaps were identified in knowledge and guideline awareness: most participants reported being insufficiently informed about national and international recommendations, with midwives and nurses showing the lowest levels of awareness (95,8% n=23, 95,2% n=20, respectively), while psychiatrists demonstrated higher knowledge (p<0.01) A majority of respondents (82.8%, n=183) reported that no clearly established clinical pathway for peripartum depression existed in their institution. Familiarity with screening methods and diagnostic criteria was limited: 80.1% (n=177) felt insufficiently informed about the appropriate timing of perinatal mental health screening.

**Conclusions:**

Most healthcare professionals recognize perinatal vulnerability and support screening, significant misconceptions, insufficient knowledge, and lack of clinical pathways persist. Our findings, although limited by sample size, underscore the need for targeted education, guideline dissemination, and structured institutional protocols to strengthen perinatal mental healthcare in Serbia. Nationwide studies with larger samples are warranted.

**Disclosure of Interest:**

None Declared

